# FACTORS ASSOCIATED WITH WANDERING AMONG PERSONS WITH DEMENTIA: A RETROSPECTIVE STUDY

**DOI:** 10.1093/geroni/igad104.1894

**Published:** 2023-12-21

**Authors:** Antonio Miguel-Cruz, Hector Perez, Emily Rutledge, Christine Daum, Lili Liu

**Affiliations:** University of Alberta, Edmonton, Alberta, Canada; University of Waterloo, Waterloo, Ontario, Canada; University of Waterloo, Waterloo, Ontario, Canada; University of Waterloo, Waterloo, Ontario, Canada; University of Waterloo, Waterloo, Ontario, Canada

## Abstract

Persons living with dementia are at increased risk of getting lost and going missing due to critical wandering. Risk factors associated with critical wandering within this population are underexplored, thus prompting this study. In this retrospective observational study, we examined anonymized data from 25,785 MedicAlert® subscribers. We used a logistic regression model with a self-reported critical wandering incident as the outcome variable (p< 0.05). The average age of our sample was 75.42 (SD 14.34). Fifty-one percent (13,064/25,785) of cases had dementia and almost 22% (5,561/25,785) were involved in a critical wandering incident at least once. People living with dementia were two and a half times more likely to be involved in a missing incident compared to people without dementia (OR=2.56, 95% CI [2.39, 2.73], p< 0.001). The likelihood of being involved in a lost incident increased with advancing age; people 95-104 years old were seven times more likely to wander than those under age 65 (OR=7.11, 95% CI [5.96, 8.47], p< 0.001). Sex at birth, official Canadian languages spoken, ethnic background, population density, living arrangement, and medication were associated with increased dementia-related missing incidents. Numerous risk factors for missing incidents were identified. Our study paves the way for implementing preventative strategies to ultimately decrease the risk of going missing for person living with dementia.

